# Persistent post-concussion symptoms include neural auditory processing in young children

**DOI:** 10.2217/cnc-2023-0013

**Published:** 2024-06-28

**Authors:** Silvia Bonacina, Jennifer Krizman, Jacob Farley, Trent Nicol, Cynthia R LaBella, Nina Kraus

**Affiliations:** 1 Auditory Neuroscience Laboratory, Northwestern University, Evanston, IL 60208, USA; 2 Department of Communication Sciences and Disorders, Northwestern University, Evanston, IL 60208, USA; 3 Department of Neurobiology, Northwestern University, Evanston, IL 60208, USA; 4 Department of Otolaryngology, Northwestern University, Chicago, IL 60611, USA; 5 Division of Orthopaedic Surgery & Sports Medicine, Ann & Robert H Lurie Children’s Hospital of Chicago, Chicago, IL 60611, USA; 6 Department of Pediatrics, Feinberg School of Medicine, Northwestern University, Chicago, IL 60611, USA

**Keywords:** auditory processing, frequency-following response, frequency encoding, post-concussion symptoms, recovery time

## Abstract

**Aim:**

Difficulty understanding speech following concussion is likely caused by auditory processing impairments. We hypothesized that concussion disrupts pitch and phonetic processing of a sound, cues in understanding a talker.

**Patients & methods/results:**

We obtained frequency following responses to a syllable from 120 concussed and 120 control. Encoding of the fundamental frequency (F0), a pitch cue and the first formant (F1), a phonetic cue, was poorer in concussed children. The F0 reduction was greater in the children assessed within 2 weeks of their injuries.

**Conclusion:**

Concussions affect auditory processing. Results strengthen evidence of reduced F0 encoding in children with concussion and call for longitudinal study aimed at monitoring the recovery course with respect to the auditory system.

Concussion among young children increasingly represents a source of concern among parents, school personnel, healthcare providers and policy makers. Over 1 million pediatric mild traumatic brain injuries (TBIs) occur annually in USA [1] and their consequences may disrupt physical and socio-emotional health, neurological function and academic achievement, even after acute symptoms resolve [2].

In recent years, a number of studies documented that concussion negatively affects auditory processing in children and adolescents, leading to alteration in speech processing like distorted speech discrimination or sound localization or difficulties in remembering and following oral instructions and/or understanding rapid or degraded speech, especially in noisy environments [3–5]. These auditory processing difficulties often affect the return to school and the learning process.

The current study on a large sample of young participants is aimed at studying concussion's effect on auditory processing using the frequency following response (FFR). The FFR, which is an objective auditory physiological measure that assesses processing of complex sounds such as speech or music [11], has identified sound processing deficits in cases of concussion [4,6–10]. Because the FFR is collected passively, it is well positioned to potentially assist in concussion assessment, monitoring and management, which otherwise are based on self-report symptoms, becoming a reliable measure to refer as indicator of concussion [12].

Previous studies highlighted a reduction in frequency encoding of speech after sustaining a concussion [4,5,7–10]. The effect is particularly salient at the fundamental frequency (F0), which is the frequency that provides the cue to the identity of a talker and is used to lock-on the pitch of the talker's voice in a noisy background [13]. Here, we explore the severity of the symptoms post-concussion, and we extend the investigation of frequency encoding to higher frequencies, namely the first formant (F1), because properly encoding the latter is crucial for learning as it allows a child to distinguish between words with similar phonemes, enabling accurate perception and understanding of a word as it unfolds in time. To test whether concussion affects both F0 and F1, we collected FFRs on children and adolescents with concussion and compare their encoding of both frequency ranges to age- and sex-matched healthy controls. Finally, we explored how post-concussion frequency encoding changes over the course of concussion recovery. We predicted both F0 and F1 would be disrupted in these children, with the greatest disruptions seen earlier in the recovery timeline. This study strengthens and expands previous findings by including participants who experienced concussion in various settings so as to increase the understanding of the occurrence of auditory processing decline at the time of injury and its recovery timeline.

## Methods

### Participants

Two groups of children (age range from 8 to 19 years) participated in this study. Inclusion criteria were normal hearing, no neurologic disease and no history of severe TBI.

The concussion group (n = 120, 46 males, mean age = 14.46 years, SD = 2.28 y) was recruited from the Institute of Sports Medicine at Ann & Robert H. Lurie Children's Hospital of Chicago, a tertiary care clinic, often seeing referrals from primary care physicians for concussions that are not resolving within 4 weeks. Flyers related to this study were posted on the waiting room. A research assistant of this study was also available in the room to introduce and explain the study to those who expressed interest. The primary physician presented the study to each family during the visit. Children in this group met clinical diagnostic criteria for a concussion and participated in the experiment following their medical evaluation by a sports medicine physician with expertise in concussion diagnosis and management. On average, they were evaluated 48 days after their injury (mean = 47.91 days, SD, 61.15 days, range: 2–344 days, median = 28). The histogram reported below (Supplementary Figure 1) represents the distribution of the number of days since concussion. To explore differences in frequency reductions depending on the time of the assessment, we divided the children with concussion in two groups, those assessed within 2 weeks after their injury (‘Early’ assessment n = 25) and those assessed after two weeks and beyond (‘Later’ assessment n = 92); for 3 children the exact time of the assessment after the injury was not available so they were not included in this particular analysis. The motivation for the cut off of the ‘Early’ group at 2 weeks comes from the observation that in about 80–90% of cases, individuals' symptoms resolve within 2 weeks in young people [14]. Injuries were attributed to sports (n = 79), moving vehicle accident (n = 32) and other activities (n = 9). Ninety-seven of the children reported a history of a previous concussion.

The control group (n = 120, 46 males, mean age = 14.64 years, SD = 2.29 years) was selected based on the age range from the laboratory database including data from past studies; none of the control participants reported a history of brain injury as reported by their families in a self-report questionnaire investigating family and personal history.

The groups were matched with respect to age (t[238] = 0.611, p = 0.432, Cohen's d = 0.0789) and had the same distribution of males and females.

To verify normal hearing before FFR testing, all participants were screened with distortion product otoacoustic emission and with the collection and online review of the click auditory brainstem response (ABR), an objective measurement of auditory pathway function from the auditory nerve to the mesencephalon [15,16]. All the subjects included in the study passed a hearing screening involving distortion product otoacoustic emission screening, suggesting normal outer hair cell function in the cochlea (>6 dB signal-to-noise ratio from 0.4–5 kHz) and normal hearing thresholds. All subjects had also normal ABR to a 100 μs click presented at 80.4 dB SPL to the right ear and the two groups had similar ABR onset response timing (wave V: t[238] = 0.449, p = 0.485, Cohen's d = 0.221). The comparable click-evoked response timing suggests that concussions do not compromise signal transduction through the peripheral auditory pathway; this observation is consistent with previous research [17].

### Neurophysiology

Frequency-following responses (FFRs) were elicited by a 40 ms sound /d/ synthesized in a Klatt synthesizer (SenSyn, Sensimetrics Corporation, MA, USA). The stimulus begins with a plosive burst during the first 10 ms, with a 5 ms voice-onset time. During the voiced period of the stimulus, the fundamental frequency (F0) rises linearly from 103 → 125 Hz while the formants shift linearly as follows: F1 220 → 720 Hz, F2 1700 → 1240 Hz, and F3 2580 → 2500 Hz. The last two formants are steady throughout the stimulus (F4 3600 Hz, F5 4500 Hz). Although the stimulus is brief and there is no vowel, it is perceived as the consonant-vowel syllable [da].

Stimuli were delivered and responses were collected through a Bio-logic Navigator Pro System (Natus Medical Inc., WI, USA). FFRs were measured in a vertical montage with three Ag-AgCl electrodes (Cz active, Fpz ground, right earlobe reference). Stimuli were delivered to the right ear in alternating polarities at 80.4 dB sound pressure level at at rate of 10.9 Hz through an electromagnetically shielded insert earphone (Etymotic Research, IL, USA). Responses were filtered online from 100–2000 Hz (second-order Butterworth) and sampled at 12 kHz. Online artifact rejection was employed at ±23 μV, and two blocks of 3000 artifact-free stimulus presentations were averaged with a 75 ms recording epoch (including a 15.8 ms non-stimulus period, which served as a control measure of background noise). While FFR was recorded passively, the participants sat in a comfortable chair in a quiet room. No behavioral response from the participant was required. The recording, including the click ABR, lasted about 40 min. The same recording procedure was used for both groups.

Children in the concussion group completed the Post Concussion Symptom Scale (PCSS) to report their symptom load. For each of the 22 symptoms, encompassing neurocognitive, emotional and somatic aspects of concussion symptomology, subjects indicated on a Likert scale of 0–6 the intensity of each symptom. The PCSS total score is the sum of the scores for each symptom and represents the subject's symptom load. Higher scores reflect greater symptom loads. Normative data, test-retest reliability (intraclass correlation coefficient [ICC], 0.62–0.69), internal consistency (r = 0.93) and minimal detectable change (MDC; total score of 12.3 points) of the PCSS have already been established [18]. Total PCSS scores at the first visit ranged from 1 to 108 (mean 37.68; SD 25.91). Among the 22 symptoms reported, one is related to the auditory area, and it falls under the category of ‘sensitivity to noise’. Seventy-two percent (86/120) of concussed children reported this symptom with an average intensity of 2.08 (SD = 1.87). The bar graph reported below (Supplementary Figure 2) represents the average score for each symptom across children, the red bar refers to the ‘sensitivity to noise’ symptom.

### Data analyses

Neurophysiological responses were analyzed with respect to F0 and F1 magnitude. The fundamental frequency (F0) amplitude was defined as the spectral amplitude between 75 and 175 Hz, F1 was computed between 175 and 750 Hz. To determine spectral amplitudes, the response (from 19.5 to 44.2 ms) was converted to the frequency domain (fast Fourier transformation with a 2 ms Hanning window). To determine a response magnitude over the prestimulus region, the root-mean-squared amplitude of the response was computed from -15.8 to 0 ms.

### Statistical analysis

A multivariate ANOVA with sex and age as covariates was run to compare all children with concussion and the control group across F0 and F1. A separate univariate ANOVA with sex and age as covariates was run to verify that the two groups were matched in their pre-stimulus root mean squares (RMS) amplitude.

To investigate the change in F0 and F1 encoding over the course of concussion recovery, we focused on the children with concussion. We performed a repeated measures ANOVA (frequency × time since injury) to test for any differences with respect to frequency reduction over time. To investigate the presence of a difference in F0 and F1 encoding between the children reporting sensitivity to noise and those who did not report it we performed another multivariate ANOVA with sex and age as covariate. All statistics reported reflect two-tailed tests, with a maximum alpha of 0.05 to assess significance.

## Results

### Children with symptoms post-concussion reveal both F0 & F1 reductions compared with age- & sex-matched controls

**Figure 1.**
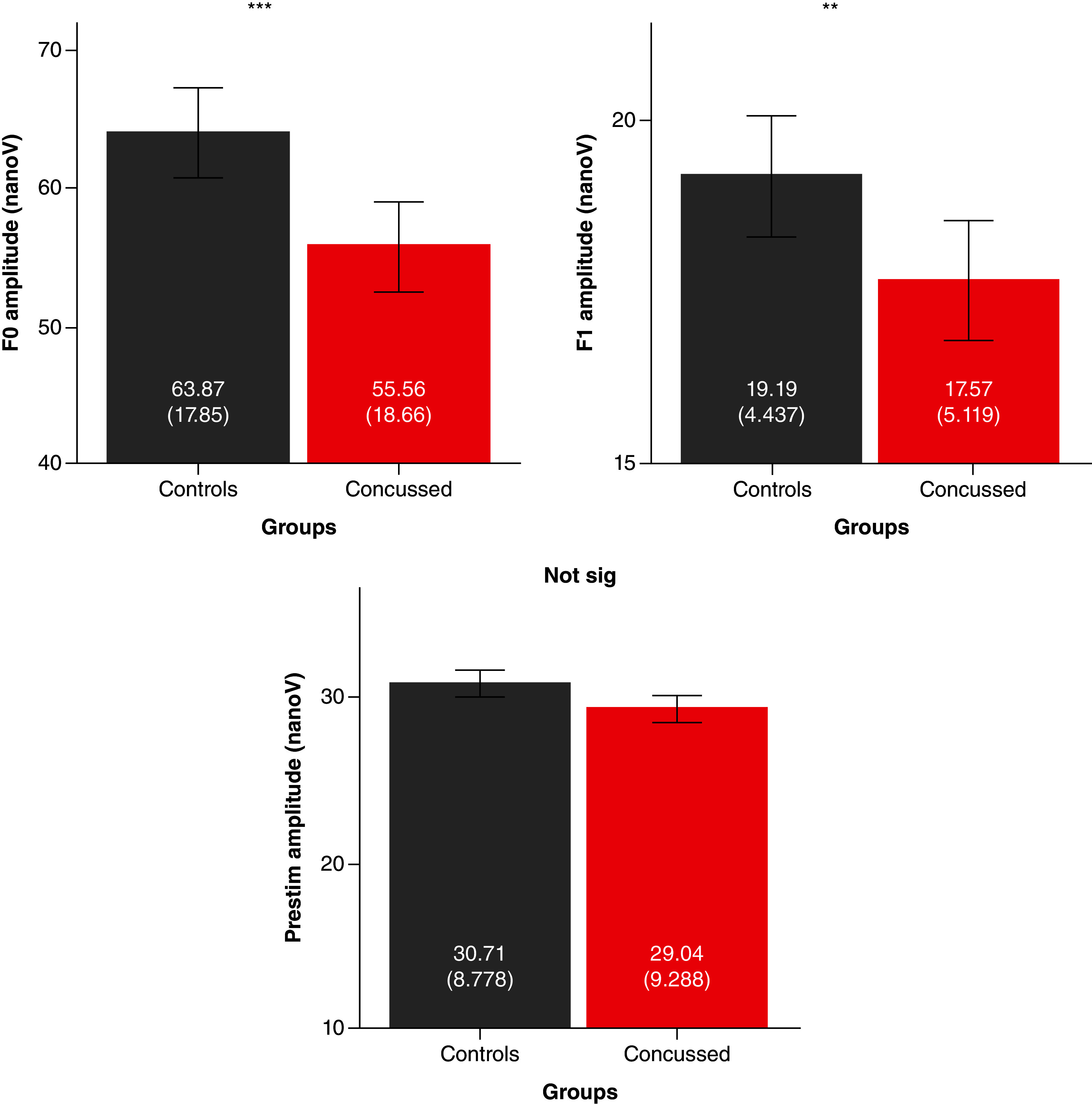
Bar graphs plot the mean ± standard errors of fundamental frequency (F0), first harmonics (F1) and prestimulus root mean squares amplitude (nanovolts) for the control (black) and concussion (red)+ groups. Higher amplitude reflects better encoding. Each group includes 120 participants. Mean and standard deviations of the amplitude (nanovolts) for each group is provided on the respective bar. Statistics were performed by Manova comparing eachfrequency following response variable between the two groups, ***p < 0.001; **p < 0.01.

**Figure 2.**
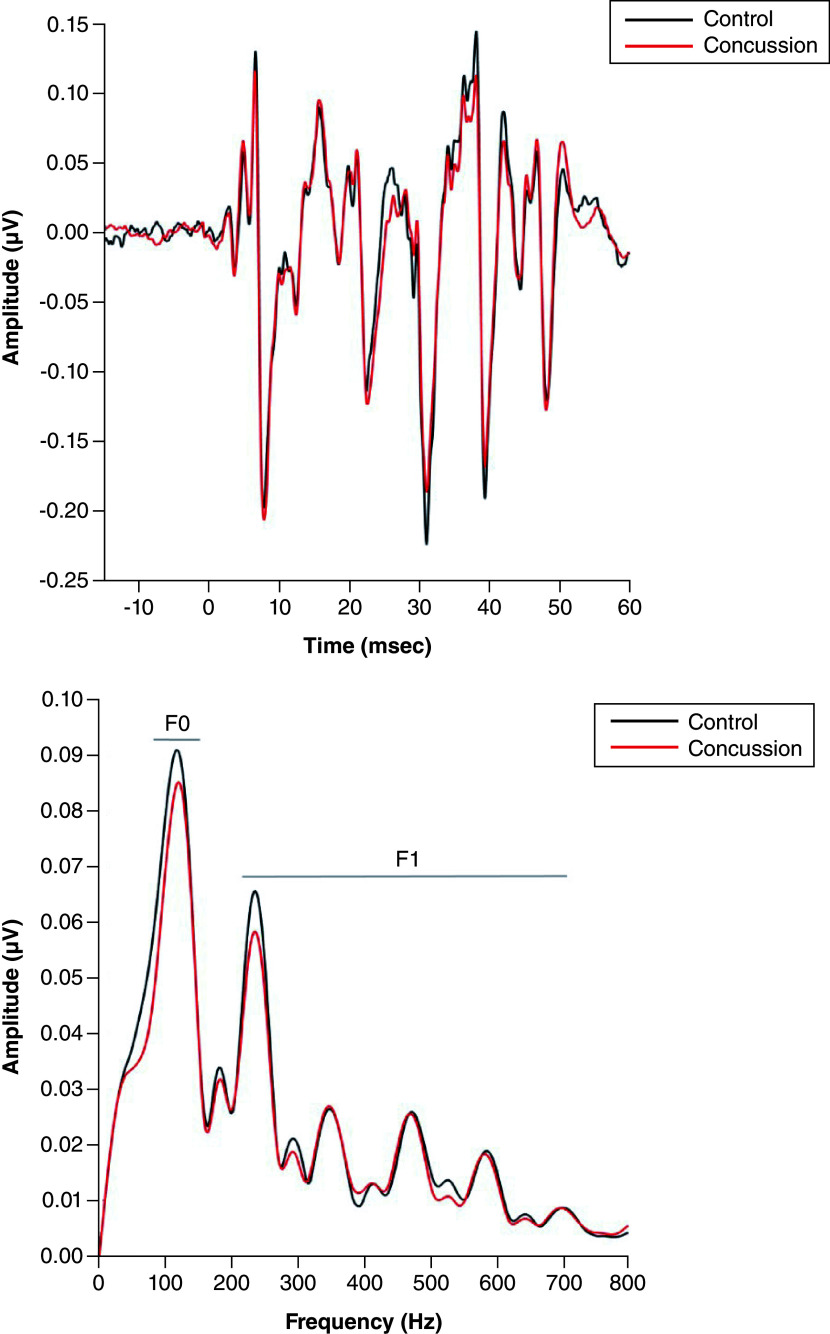
Grand averages of frequency following responses in the time and frequency domains. Black line refers to the control group while red line refers to the concussion group.

The children who sustained a concussion differ from controls across both frequencies (F_(2, 235)_ = 6.426, p = 0.002, η^2^ = 0.52). In particular, children with concussion reveal smaller responses to the F0 (F_(1, 239)_ = 12.342, p < 0.001, η^2^ = 0.50) and F1 (F_(1, 239)_ = 5.824, p = 0.017, η^2^ = 0.24) than their peers. No difference is present with respect to neural background noise (F_(1, 239)_ = 1.771, p = 0.185, η^2^ = 0.007). Bar graphs plot of F0, F1 and neural background noise for the control and the concussion groups are shown in Figure 1. Grand averages of FFRs in the time and frequency domains for the Control and the Concussion groups are shown in Figure 2.

### The F0 & F1 reductions in children after concussions develop distinctly over time

**Figure 3.**
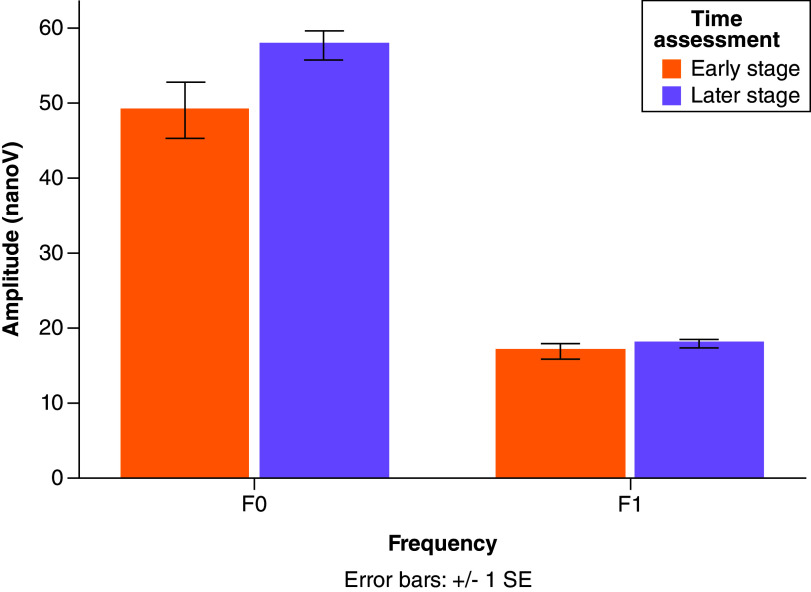
Bar graph compares the encoding of both fundamental frequency (F0; left) and first harmonics (F1; right) between children assessed during the first two weeks after their injury (early stage; n = 25; orange) and those assessed at a later stage (later stage; n = 92; purple). Higher amplitude reflects better encoding. Mean and standard deviations of the amplitude (nanovolts) for each group is provided on the respective bar.

The F0 reduction is overall larger than the F1 reduction (F_(1, 113)_ = 19.258, p < 0.001, η^2^ = 0.146). Moreover, the F0 reduction is most apparent in the children assessed during the first 2 weeks after a concussion (1–14 days) than later in the post-concussion course of recovery (15–344 days) whereas the F1 reduction does not reveal the same Frequency by Time interaction (F[1, 113] = 3.924, p = 0.050, η^2^ = 0.034). Bar graphs plot of F0 and F1 between children assessed at early and later stages are shown in Figure 3.

### The F0 & F1 magnitude encoding did not differ between children reporting or not reporting sensitivity to noise

The F0 and F1 encoding did not differ between children reporting (from 2 to 6) or not reporting sensitivity to noise (0 or 1) in the post concussion symptoms scale (F (1, 116) = 0.235, p = 0.791, η^2^ = 0.004).

## Discussion

The encoding of both F0 and F1 of speech was reduced in children and adolescents who experienced a concussion and reported symptoms post-concussion compared with age- and sex-matched healthy controls. All participants included in the concussion group were symptomatic at the time of the assessment and a majority reported mild sensitivity to noise among other symptoms related to neurocognitive, emotional and somatic areas.

By dividing the children in the concussion group based on the number of days after their actual injury, we found different trends between F0 and F1. F0 reduction was most reduced in children assessed at early stages after their injury than in those assessed at later stages. F1 reduction appeared to be stable over time for both groups. Even though only longitudinal data will be able to confirm these trajectories, the different trends of F0 and F1 indicate the extensive impact of concussion on auditory processing with respect to both pitch and phonetic processing in young people.

The comparison across both F0 and F1 encoding between the children reporting or not sensitivity to noise did not reveal any difference. It would be important to investigate deeper, especially given the indefiniteness of a single item related to the auditory system present in the post-concussion symptom scale. In addition, the absence of this result can be due to the fact that the FFR is very sensitive to small processing deficits that are indicative of a change at the brain level even if they are not overtly causing symptoms. In fact, detecting small variations is crucial especially when thinking about the repetitive sub-concussions that may happen when practicing contact sports that may end up in repetitive small changes that sum up in big variations.

Together, these results call for the inclusion of the auditory system among the areas of assessment, diagnosis and management of concussion.

A major limitation of this study is that we are not considering the total numbers of years of sport practice, especially with respect to the control group. Putative subconcussion, inferred from participation in contact or collision sports, also affects on auditory processing [19]. Given this, further studies should consider the impact of history of contact sport participation in both the concussed and control children.

Further studies are also needed to determine whether the reduction in both F0 and F1 is restricted to this age group, or also occurs in younger or older populations, and whether F0 and F1 return to normal levels once concussion symptoms are fully resolved. Behavioral testing for discrimination thresholds may also be interesting to include in future research. Another aspect to explore is the injury mechanism of concussion. In this study we included participants with a broad variety of injury mechanisms, as the aim was to understand whether there is a general impact of head injury on frequency encoding in children. Further studies might consider exploring differences between sport-related and other types of concussion, and even across different sports.

## Conclusion

Overall, the results of this study reinforce the growing body of work indicating that auditory processing is impacted in children with symptoms post-concussion, and its effect extends to both pitch and phonemic processing of speech in children and adolescents. The trajectory of the frequency reduction differed across the two frequencies ranges. Further longitudinal studies are needed to confirm this result by monitoring the concussion recovery course over time, ideally including a wide range of participants in terms of age and contact level of sports and testing concussed subjects after complete resolution of all symptoms in order to identify any residual deficits in auditory processing. Future studies should also examine how these deficits track with speech-recognition difficulties in concussed children, and the potential effects on social-emotional function and learning.

**Summary points**

- Concussion reduces both pitch and phonemic processing of speech in children.
- Pitch processing recovers over time while the phonemic processing deficit remains.
- Results call for the inclusion of the auditory system among the areas of assessment, diagnosis and management of concussion.

## Supplementary Material




