# Supplementary figures and images for: Persistent post-concussion symptoms include neural auditory processing in young children
Source: Concussion. 2024 Jun 28;9(1):CNC114. doi: 10.2217/cnc-2023-0013 (PMC11270634; doi:10.2217/cnc-2023-0013)

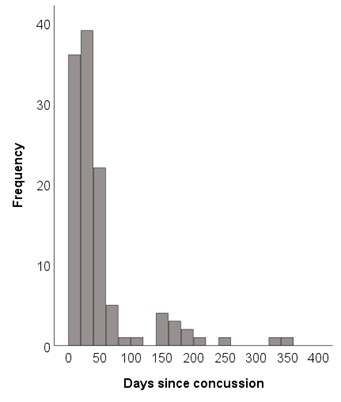

Supplement: Supplementary file 1 [file cnc-09-114-s1.jpg]

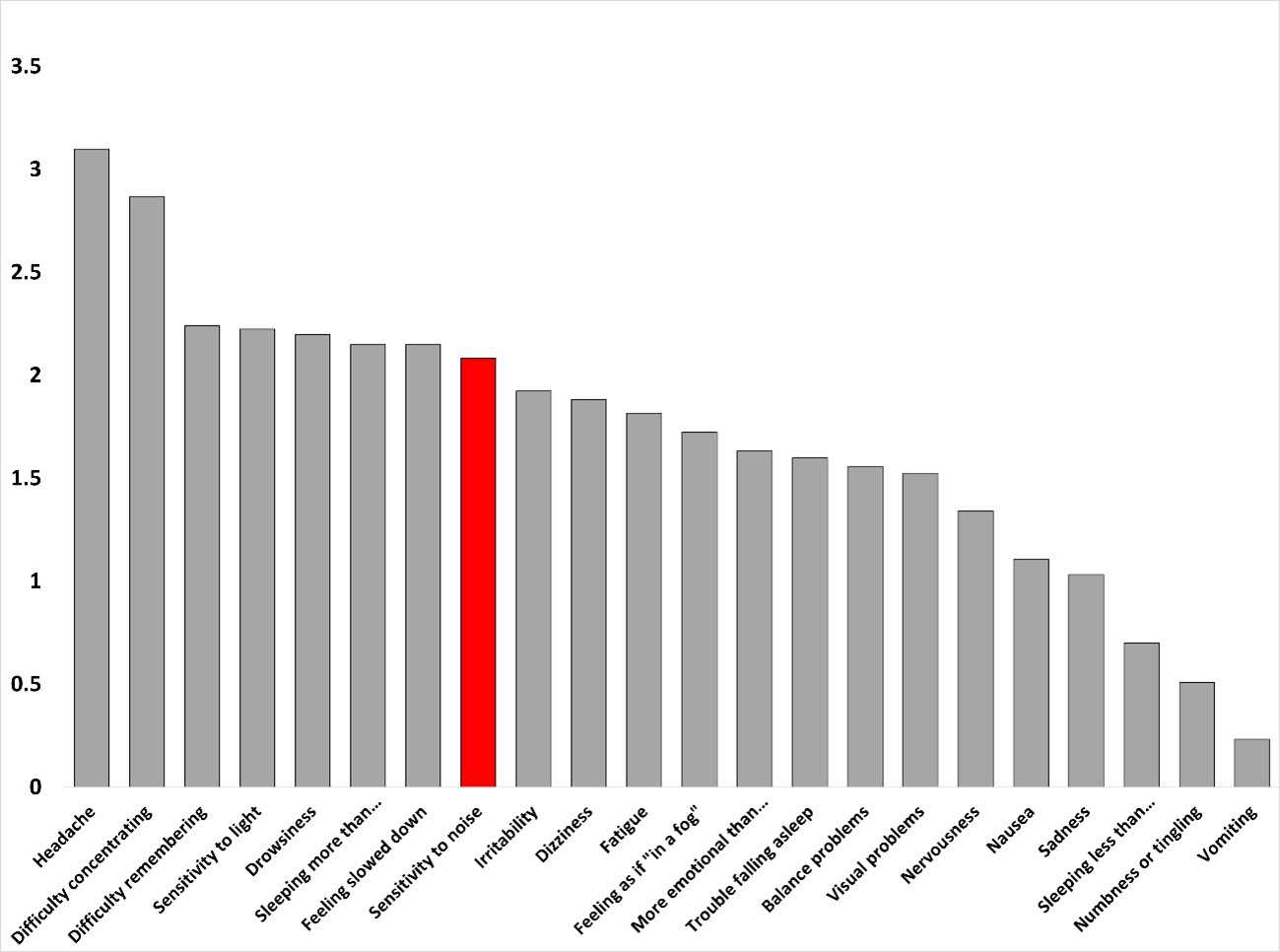

Supplement: Supplementary file 2 [file cnc-09-114-s2.jpg]
